# Nonsurgical Management of Apical Root Perforation Using Mineral Trioxide Aggregate

**DOI:** 10.1155/2021/5583909

**Published:** 2021-03-24

**Authors:** Omar Alzahrani, Faisal Alghamdi

**Affiliations:** ^1^Department of General Dentistry, The University Dental Hospital, King Abdulaziz University, 80209 Jeddah 21589, Saudi Arabia; ^2^Department of Oral Biology, Faculty of Dentistry, King Abdulaziz University, 80209 Jeddah 21589, Saudi Arabia

## Abstract

This study illustrates a conservative approach to nonsurgical management of apical root perforation in maxillary first molars. A patient was referred for retreatment of a maxillary left first molar. Her chief complaint was dull pain while biting in her maxillary left first molar. Periapical radiography showed radiolucency related to the mesiobuccal root and overextended gutta-percha through a perforation in the apical part of the distobuccal root. A CBCT scan was acquired and revealed the location and size of the apical perforation. The clinical examination showed that the tooth has been endodontically treated and the canals were filled, tender to percussion and palpation. Thus, the nonsurgical root canal retreatment was done and the perforation site was repaired by using mineral trioxide aggregate (MTA). At the one-year follow-up, after the management of apical root perforation, we observed periapical tissue healing and no pain due to percussion and palpation, without any clinical/radiological signs or symptoms. The prognosis of this case has a higher success rate with the development of new materials such as MTA. The MTA not only can seal the site of the perforation but also has the ability to induce calcification. Many factors can contribute to the success rate of perforated cases, including time, size, and location of the perforation. With the use of this material and good tools like a microscope, there are those with having higher chances of repair and eventually higher success rates.

## 1. Introduction

According to the glossary of endodontic terms, perforation is defined as the mechanical or pathological communication between the root canal system and the external tooth surface. It is one of the endodontic complications during root canal treatment. It may occur during any stage of the treatment, access cavity, cleaning and shaping, or as a result of internal resorption that is extended to the periapical tissues [1]. Eventually, chronic inflammation will occur in the periodontium, which is characterized by the formation of granulation tissue and loss of bone attachment around the perforation [2]. Currently, two treatment modalities are available for the management of these perforations, either surgical or nonsurgical approaches [3]. Many materials have been used for the treatment of root canal perforation such as amalgam, Super EBA, and glass ionomer, but recently mineral trioxide aggregate (MTA) [4], and bioceramic putty showed better results and outcomes [5]. The current case report demonstrates nonsurgical repair of root perforation on the apical part of the upper left first molar using MTA.

MTA is composed of tricalcium silicate, tricalcium aluminate, tricalcium oxide, and silicate oxide, and also, bismuth oxide was added as a radiopacifier [6, 7]. It has been widely used in endodontics for many different treatments other than perforation like direct pulp capping, retrograde filling, and apexification. Moreover, it has several advantages including biocompatibility, bacteriostatic, good sealing ability, and ability to set up in the presence of moisture [8–10].

## 2. Case Presentation

**Figure 1:**
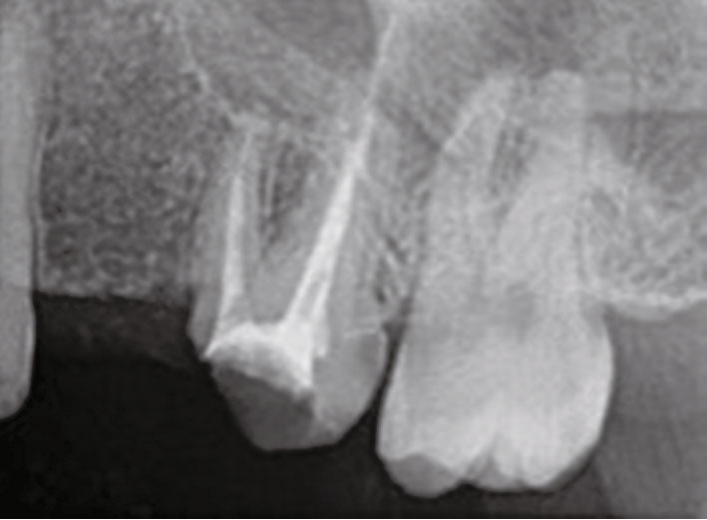
Preoperative radiograph. The intraoral periapical radiograph showed radiolucency at the root apex and overextended gutta-percha through a perforation in the apical part of one of the three roots of the maxillary left first molar.

A 27-year-old female patient reported to the Department of Endodontics at the Dental Hospital, King Abdulaziz University, complaining of dull pain while biting at her upper left first molar (#26). After clinical examination, the tooth has been endodontically treated and the canals were filled. The tooth typically did not respond to thermal or electric pulp testing, but the tooth was tender to percussion and palpation. In addition, the robing depth of the tooth was within the normal limit (2 mm). After radiographic examination, the tooth showed radiolucency related to the mesiobuccal root and overextended gutta-percha through a perforation in the apical part of the distobuccal root (Figure 1). Based on these clinical and radiographic examinations, the final diagnosis was previously treated with symptomatic apical periodontitis. Thus, the final treatment procedure was endodontic retreatment and perforation repair using MTA and restored with a final composite restoration.

**Figure 2:**
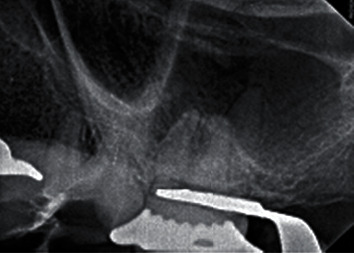
The maxillary left first molar after removal of the gutta-percha from the three canals.

**Figure 3:**
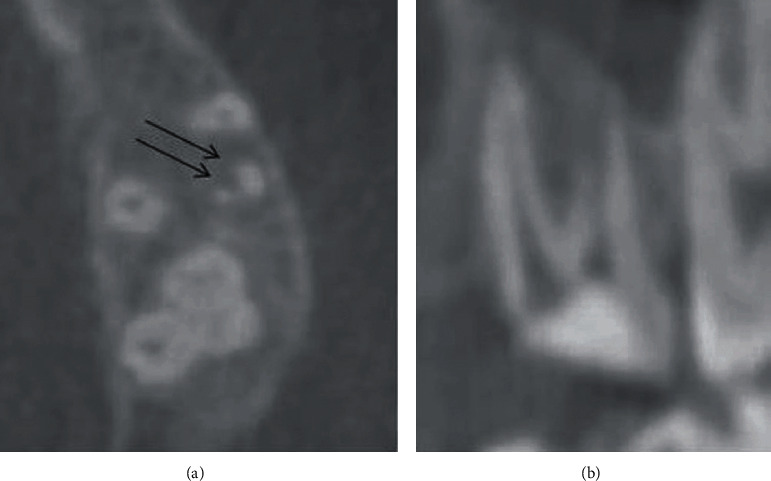
Preoperative CBCT images. The axial view of CBCT showed the apical perforation site (black arrows) at the distobuccal root (a). The sagittal view of CBCT showed the apical perforation site of the tooth apex.

A thorough explanation was given to the patient regarding the treatment procedure. The patient gave her written informed consent, in accordance with the Helsinki Declaration on the Ethical Principles for Medical Research Involving Human Subjects for the publication of her case report. Anesthesia was given using one carpule of 4% articaine with 1 : 100,000 epi as buccal infiltration. The rubber dam was applied and caries excavation using a size 3 mm in diameter round diamond bur, and the access cavity was edited using the conic steel bur endo-Z (Dentsply Maillefer, Ballaigues, Switzerland) at high-speed underwater spray; Reciproc® blue (R25/0.08 mm) (VDW GmbH, Munich, Germany) was used for the removal of the gutta-percha and three hand H-files (Dentsply Maillefer, Ballaigues, Switzerland) for the removal of the overextended gutta-percha in the distobuccal root (Figure 2). Cavit™ temporary filling material (3M ESPE, Seefeld, Germany) was then placed, and the rubber dam was removed, and the patient was asked to take cone-beam computed tomography (CBCT) to determine the location and size of the apical perforation (Figures 3(a) and 3(b)).

**Figure 4:**
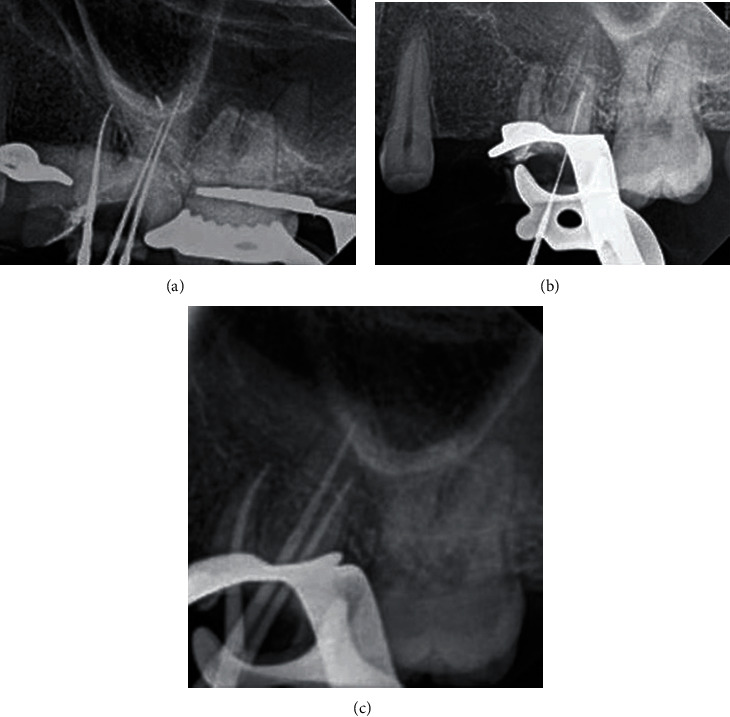
Intraoral periapical radiograph for the treatment procedure of maxillary left first molar. Working length was determined (a). Placement of MTA to repair the perforation site at the distobuccal root (b). Matching cone for the three canals after cleaning and shaping was done (c).

**Figure 5:**
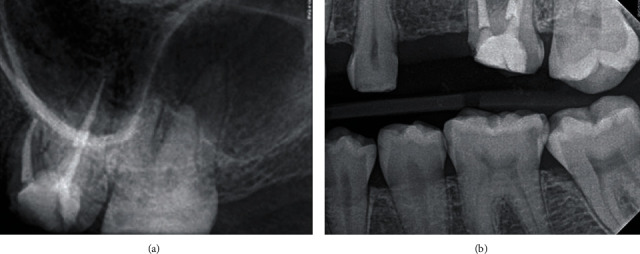
Postoperative intraoral radiographs. Intraoral periapical radiograph of the maxillary left first molar after root canal retreatment (a). Intraoral bitewing radiograph of the maxillary left first molar after root canal retreatment (b).

The second visit after CBCT was made, determining the working length using a DentaPort ZX apex locator (J. Morita Mfg. Corp., Kyoto, Japan), then radiographic X-ray for the final working length was taken (Figure 4(a)), and also, cleaning and shaping were done using Vortex blue (#40/0.04 mm) (Dentsply Sirona, Charlotte, USA) for all canals along with chlorhexidine as an irrigant. Using the MAP system (Produits Dentaires, Vevey, Switzerland) under a high magnification ZEISS EXTARO-300 dental microscope (Carl Zeiss Meditec AG, Berlin, Germany), the ProRoot MTA (Dentsply Tulsa Dental, Tulsa, OK, USA) was placed in the apical third of the canal (Figure 4(b)), and a small size gutta-percha was placed in the main canal to prevent closure, the temporary filing was applied, and the patient was dismissed to allow the setting of MTA material. The third visit followed the same anesthesia and rubber dam isolation as explained previously, removal of the temporary filling and irrigation using 15 mL of 3% NaOCl activated with the EndoVac® irrigation system (Vista Dental, Racine, WI, USA) for 5 minutes in each canal and using a 30-gauge side vented irrigating needle (Max-I-Probe, Dentsply Maillefer, Ballaigues, Switzerland). After that, all canals were dried using an absorbent paper point size 30 blue color code (Meta Dental Corp., New York, USA) and master cones were fitted (Figure 4(c)). Cold single cone technique (hydraulic condensation technique) with (#30/0.06) matching gutta-percha cones and bioceramic sealer were used as an obturation method. Total Fill® bioceramic sealer (TotalFill BC Sealer) (FKG Dentaire, La Chaux-des-Fonds, Switzerland) was applied in each canal. Then, these matching cones were cut and compacted using an appropriate Buchanan hand plugger size #2 (SybronEndo Corporation, Orange, CA, USA) at the level of the orifice. The tooth was restored using dental glass ionomer filling material (GC FUJI IX GP) (GC Corporation, Tokyo, Japan), the bite was checked, and postoperative radiographs were taken (Figures 5(a) and 5(b)). Postoperative instruction was given to the patient and prescription of analgesics 600 mg three times a day for three days. After a week from the visit, the dental glass ionomer filling material was removed and replaced by final bulk-fill composite restoration (SureFil®SDR®Flow, Dentsply, York, PA, USA) applied according to the manufacturer's instructions.

**Figure 6:**
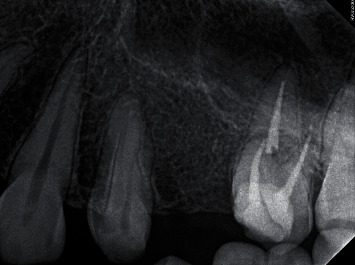
Postoperative intraoral periapical radiograph of the maxillary left first molar after a one-year follow-up period of retreatment: the radiolucent region of the tooth was healing.

The 1-year follow-up revealed periapical tissue healing at the root apex of the upper left first molar (#26) (Figure 6). There is no pain due to percussion and palpation, and no mobility of the tooth. The clinical and radiological examinations revealed the progression of apical root perforation of tooth #26. It was stable, aesthetically please state of the tooth with no pathological findings or unpleasing problems observed. Further yearly recall visits were scheduled to monitor the progress of tooth #26.

## 3. Discussion

Many factors affect the prognosis of root perforation, including the location, size, and time of the contamination. The location of the apical perforation has a good prognosis [4]. Another factor that may influence the treatment prognosis is the size of the perforation; some authors suggested in large perforations, the use of an internal matrix will help to prevent the extrusion of the material to the periradicular tissues [11]. In the present case, the apical perforation is 3 mm in size, and there was no need for such a matrix. Time plays a critical role in the prognosis of the treatment [12]. However, in this case, perforation happened two years ago, which may decrease the prognosis, and the perforation can be considered as contaminated; this kind of perforation might have worse repair when compared with noncontaminated [13].

The material contributes to the success of the perforation repair [14]. An ideal material should have the ability to induce the formation of bone and biocompatible to the periradicular tissues, maintain the sealing in the perforation site, nonresorbable, and does not dissolve in the tissue fluid [15]. In the past, many materials were used to repair the perforation including amalgam, composite, and glass ionomers. Currently, MTA has superior properties and sealing ability when compared to different materials. It also has a high pH of around 12.5, which promotes new tissue formation and repair [16]. MTA has the ability to release calcium since it has calcium silicate which is one of its components that are believed to increase the sealing ability of the material [17].

In a study done by Santos et al. [18] in 2021, they have evaluated the biocompatibility of TotalFill BC Sealer (FKG, La Chaux-des-Fonds, Switzerland) and TotalFill BC Sealer HiFlow (FKG, La Chaux-des-Fonds, Switzerland) through subcutaneous implantation in the connective tissue of rats. They were concluded in their study; both bioceramic sealers were biocompatible and showed potential bioactivity compared to the AH Plus sealers. This is in agreement with our findings when injected TotalFill BC Sealer in the root canals of tooth #26 and given appropriate periapical lesion healing and improved the obturation quality. In another study done by Muedra et al. [19] in 2021, they compared dentinal penetration between EndoSequence bioceramic sealer and BioRoot root canal sealer. All the root canals were obturated using a single-cone technique in their study. They were concluded by dentinal penetration significantly higher for EndoSequence bioceramic sealer compared to BioRoot root canal sealer. McMichael et al. [20] have shown that bioceramic sealers penetrated tubules as deep as 2 mm when a single cone technique was applied. Jeong et al. [21] have also shown that penetration of a tricalcium silicate sealer into the dentinal tubules happened independently of the obturation technique, a finding that is in accordance with our study, in which TotalFill BC Sealer penetrated tubules deeply and using a single cone technique to assist in the healing of apical periodontitis and improve the obturation quality of the tooth #26.

Finally, it can be concluded that the criteria for success of perforated teeth include the absence of the patient's symptoms, no pain due to percussion and palpation, and no mobility of the tooth. Moreover, there is radiographic evidence of bone formation or calcification at the site of perforation [22]. Also, the type of sealer can play an important role in improving the obturation quality of the root canals.

## 4. Conclusions

The prognosis currently has a higher success rate with the development of new materials such as MTA. The MTA not only can seal the site of the perforation but also has the ability to induce calcification. Many factors can contribute to the success rate of perforated cases, including time, size, and location of the perforation. With the use of this material and good tools like a microscope, there are those with having higher chances of repair and eventually higher success rates.

## Data Availability

The data supporting this study can be accessed by readers freely through the availability of the same by the authors, as long as the patient's personal data is preserved. Images can be requested and notes from the medical record can also be viewed at any time, as long as there is no identification of the patient, as previously stated.
